# Supplementary material for: Exploring the nature of collisionless shocks under laboratory conditions
Source: Sci Rep. 2014 Feb 3;4:3934. doi: 10.1038/srep03934 (PMC3910018; doi:10.1038/srep03934)
Supplement: Supplementary Information — Supplementary Info File #1 [file srep03934-s1.pdf]

Exploring the nature of collisionless shocks under laboratory conditions

A. Stockem, F. Fiuza, A. Bret, R. A. Fonseca, and L. O. Silva

Dispersion relation of electromagnetic modes

The relativistic distribution function

$$f_{re} = n_{r0} \begin{cases} \exp\left\{-\mu\left[\gamma_0\left(\gamma-\varphi\right)-1+u_0\sqrt{(\sqrt{1+u_x^2}-\varphi)^2-1}\right]\right\} & u_x < -u_c \\ \exp\left\{-\mu\left[\gamma_0\gamma_\perp-1\right]\right\} & |u_x| \leq u_c \\ \exp\left\{-\mu\left[\gamma_0\left(\gamma-\varphi\right)-1-u_0\sqrt{(\sqrt{1+u_x^2}-\varphi)^2-1}\right]\right\} & u_x > u_c \end{cases} \quad (1)$$

with $\gamma = \sqrt{1+u_x^2+u_y^2+u_z^2}$, $\gamma_\perp = \sqrt{1+u_y^2+u_z^2}$, $\gamma_c = 1+\varphi$ and $u_c = \sqrt{\gamma_c^2-1}, \beta_c = \sqrt{2\varphi}$ for $\varphi \ll 1$ and

$$n_{r0} = \frac{\gamma_0^2\mu^2}{2\pi e^\mu}\left[2u_c(1+\gamma_0\mu)e^{-\mu\gamma_0} + e^{\mu\gamma_0\varphi}\sum_\pm \int_{g_c}^\infty d\gamma \frac{\gamma(1+\mu\gamma_0\gamma)}{\sqrt{\gamma^2-1}} e^{[-\mu(\gamma_0\gamma\pm u_0\sqrt{(\gamma-\varphi)^2-1})]}\right]^{-1} \quad (2)$$

is used to calculate the dispersion relation

$$k^2c^2 - \omega^2 - \omega_{pe}^2(U_e+V_e) = 0 \quad (3)$$

with the definitions

$$U_e = \int_{-\infty}^{\infty} d^3u \frac{u_x}{\gamma}\frac{\partial f_{re}}{\partial u_x} \quad (4)$$

and

$$V_e = \int_{-\infty}^{\infty} d^3u \frac{u_x^2}{\gamma\left(\gamma\frac{\omega}{kc}-u_z\right)}\frac{\partial f}{\partial u_z} \quad (5)$$

where only fluctuations $\mathbf{k} = k\mathbf{e_z}$ perpendicular to the fluid velocity $\mathbf{u_0} = u_0\mathbf{e_x}$ were considered. The parameters were derived to

$$U_r = -n_{r0}2\pi\mu e^{\mu(\gamma_0\varphi+1)}\sum_\pm \int_{\gamma_c}^\infty \sqrt{\gamma^2-1}e^{\mp\mu u_0\sqrt{(\gamma-\varphi)^2-1}} \times \left[\gamma_0\gamma\Gamma\left(0,\mu\gamma_0\gamma\right) \pm \frac{\beta_0}{\mu}\frac{\gamma-\varphi}{\sqrt{(\gamma-\varphi)^2-1}}e^{-\mu\gamma_0\gamma}\right] \quad (6)$$

and

$$V_r = n_{r0}4\mu\gamma_0\int_0^\infty du_z u_z^2 \int_0^\infty du_y \left[\frac{2}{\gamma_\perp}e^{-\mu(\gamma_0\gamma_\perp-1)}\int_0^{u_c} du_x \frac{u_x^2}{\gamma(\gamma^2y^2+u_z^2)} + \sum_\pm e^{\mu(\gamma_0\varphi+1)}\int_{u_c}^\infty \frac{u_x^2}{\gamma^2(\gamma^2y^2+u_z^2)}e^{-\mu(\gamma_0\gamma\pm u_0\sqrt{(\sqrt{1+u_x^2}-\varphi)^2-1)}}\right] \quad (7)$$

and solved numerically for the general case.
